# Longitudinal decline in lung function: a community-based cohort study in Korea

**DOI:** 10.1038/s41598-019-49598-9

**Published:** 2019-09-20

**Authors:** Ah Young Leem, Boram Park, Young Sam Kim, Joon Chang, Sungho Won, Ji Ye Jung

**Affiliations:** 1 Division of Pulmonology, Department of Internal Medicine, Institute of Chest Disease, Severance Hospital, Yonsei University College of Medicine, Seoul, Republic of Korea; 2 Department of Epidemiology and Biostatistics, School of Public Health, Seoul National University, Seoul, Korea

**Keywords:** Chronic obstructive pulmonary disease, Epidemiology

## Abstract

Progressive decline in lung function is the hallmark of chronic obstructive pulmonary disease (COPD). We aimed to assess the rate of decline in forced expiratory volume in 1 second (FEV_1_) in patients from a community cohort database in Korea. 5,865 subjects aged 40–69 years from the Ansung-Ansan cohort database I–III (2001–2006) were included in this study. We assessed the annual rate of decline in FEV_1_ over time in relation to smoking status, patient sex, and presence or absence of pre-bronchodilator airflow limitation using a generalized additive mixed model. The mean follow-up duration was 3.8 years. The annual mean decline in FEV_1_ in the entire cohort was significantly more rapid for men than women (31.3 mL vs 27.0 mL, *P* = 0.003). Among men without pre-bronchodilator airflow limitation, annual mean declines in FEV_1_ were 31.5, 35.5, and 40.1 mL for never smokers, former smokers (*P* = 0.09 vs. never smokers), and current smokers (*P* < 0.001 vs. never smokers), respectively; and 23.4, 19.7, and 33.9 mL, respectively, for men with pre-bronchodilator airflow limitation. Thus, among Korean males, smoking accelerates lung function decline over time whereas smoking cessation slows the rate of FEV_1_ decline regardless of pre-bronchodilator airflow limitation. This underscores the importance of smoking cessation in Koreans.

## Introduction

Chronic obstructive pulmonary disease (COPD) is a leading cause of morbidity, mortality, and increased medical expenses worldwide^1,2^. Currently, COPD is the fourth leading cause of death and is predicted to become the third leading cause of death by 2030^3^. Fletcher and Peto reported that exposure to tobacco smoke accelerates the age-related decline in lung function as assessed by the forced expiratory volume in 1 second (FEV_1_), leading to clinical disease^4^. Since then, several population studies have supported this causal link, and therapeutic clinical trials have targeted reductions in the rapid decline in FEV_1_^3,5–10^. However, observed declines in FEV_1_ in these trials and in observational cohorts of patients with COPD have been variable, particularly among patients with severe airflow limitation^3,5–14^.

Cigarette smoking is the most important risk factor for acceleration of lung function decline^15^. Lung damage in smokers is characterized by inflammation and airway remodelling leading to airflow limitation and destruction of the lung parenchyma^16^. The underlying lung characteristics may vary; moreover, smokers differ in their susceptibility to emphysema or bronchitis^17,18^. The strongest and most consistent detrimental effect from smoking has been found in current smokers and has been shown to decrease over time after smoking cessation^15,19^.

However, COPD results from a complex interaction between genes and the environment. Cigarette smoking is the leading environmental risk factor for COPD, but <50% heavy smokers develop COPD during their lifetimes^20^. Genetic factors, age, sex, and exposure to particles are also risk factors^21^. Furthermore, any factor that affects lung growth has the potential to increase an individual’s risk of developing COPD. In previous studies, the birth weight and FEV_1_ in adulthood were positively associated, and factors in early life seem to be as important as heavy smoking for the prediction of lung function in adult life^22^.

A progressive decline in lung function has been considered the hallmark of COPD. It is also possible that a normal decline in FEV_1_ could lead to COPD. A recent study by Lange *et al*. analysed three different longitudinal cohorts and reported that approximately 50% of patients developed COPD because of an accelerated decline in FEV_1_ over time, while the other 50% developed COPD because of abnormal lung development and growth^23^. Relatively few studies evaluating the decline in lung function in healthy subjects have been reported in Asia^24^. Therefore, the present study aimed to evaluate the rate of decline in FEV_1_ in patients selected from a community cohort database in Korea.

## Results

### Baseline characteristics

**Figure 1:**
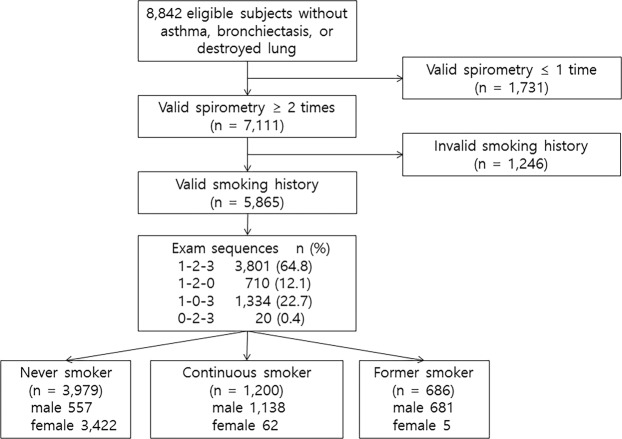
Flow chart showing the patient selection process for the study.

Of the initial 8,842 participants in the Ansung-Ansan cohort, 7,111 (80.4%) underwent at least two pulmonary function tests and were included in this study. Valid data for the smoking history were available for 5,865 of the 7,111 subjects, revealing 3,979 never smokers, 686 former smokers, and 1,200 current smokers (Fig. 1). The mean (standard deviation) follow-up duration was 3.8 (0.7) years. Table 1 shows the distribution of subjects in the different groups stratified by sex, lung function, and the smoking status.

**Table 1:** Distribution of subjects in different groups categorized by sex, lung function and smoking status.

Smoking status	Men		Women	
	Healthy	OLD	Healthy	OLD
Never smoker	521	36	3,297	125
Former smoker	595	86	5	0
Current smoker	968	170	52	10
Total	2,084	292	3,354	135

Abbreviations: OLD, obstructive lung disease.

Baseline characteristics of the study population are shown in Table 2. Of the 5,865 subjects, 2,376 (40.5%) were men and 3,489 (59.5%) were women. The proportions of male and female never smokers were 23.4% and 98.1%, respectively, while those of male and female current smokers were 47.9% and 1.8%, respectively. The mean absolute FEV_1_ value was 3.4 L for men and 2.5 L for women. Over 80% subjects were followed up for 4 years.

**Table 2:** Baseline characteristics of patients in the Ansung-Ansan cohort who had valid smoking history data (n = 5,865).

Characteristics	Men (n = 2,376)	Women (n = 3,489)
Age, years	51.0 ± 8.4	52.3 ± 8.8
Height, cm	167.1 ± 5.7	154.0 ± 5.5
Weight, kg	68.3 ± 9.4	59.1 ± 8.3
BMI, kg/m^2^	24.4 ± 2.8	24.9 ± 3.2
**Smoking status**		
Never smoker	557 (23.4)	3,422 (98.1)
Former smoker	681 (28.7)	5 (0.1)
Current smoker	1,138 (47.9)	62 (1.8)
**Lung function**		
FEV_1_, L	3.4 ± 0.6	2.5 ± 0.5
FEV_1_, % of the predicted value	107.0 ± 15.1	117.0 ± 17.3
FVC, L	4.3 ± 0.7	3.1 ± 0.6
FVC, % of predicted value	102.6 ± 13.2	107.5 ± 14.8
FEV_1_/FVC ratio, %	78.4 ± 7.9	81.7 ± 6.4
**Follow-up duration**		
2 years	383 (16.1)	486 (13.9)
4 years	1,993 (83.9)	3,003 (86.1)

Notes: Data are presented as numbers (%) or means ± standard deviations.

Abbreviations: BMI, body mass index; FEV_1_, forced expiratory volume in 1 second; FVC, forced vital capacity.

### Rate of decline in FEV_1_ in healthy never smokers

**Figure 2:**
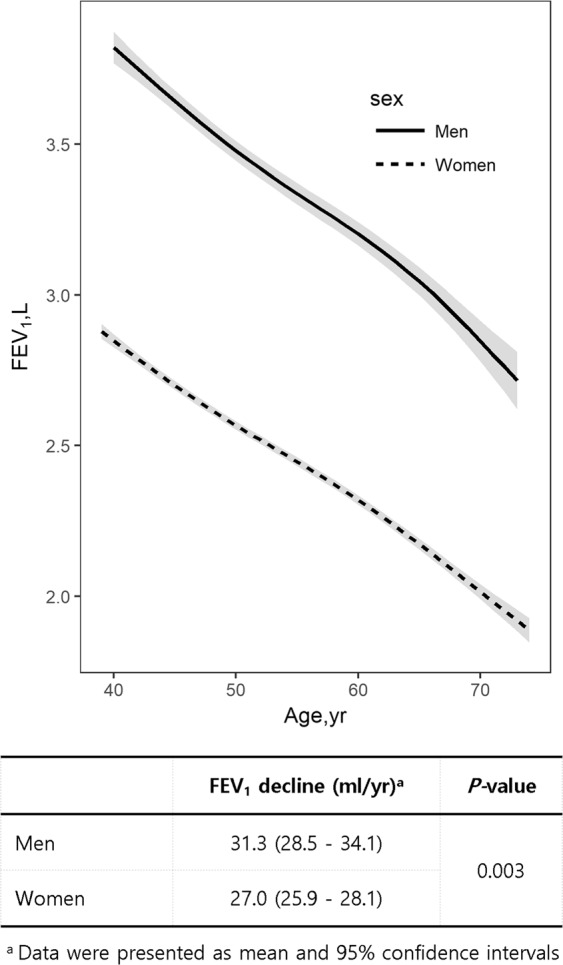
Comparison of the annual mean decline in FEV_1_ for healthy male (n = 521) and female (n = 3,297) never smokers. The annual mean decline in FEV_1_ is 31.3 mL (95% CI, 28.5–34.1) for men and 27.0 mL (95% CI, 25.9–28.1) for women (*P* = 0.003). Abbreviations: CI, confidence interval; FEV_1_, forced expiratory volume in 1 second.

Figure 2 shows the decline in FEV_1_ with age in male and female never smokers without obstructive lung disease (OLD). Men exhibited higher absolute FEV_1_ values than did women at all time points. The annual rate of decline was significantly more rapid for male healthy never smokers (31.3 mL; 95% confidence interval [CI], 28.5–34.1) than for their female counterparts (27.0 mL; 95% CI, 25.9–28.1; *P* = 0.003).

### Effect of smoking on the decline in lung function

**Figure 3:**
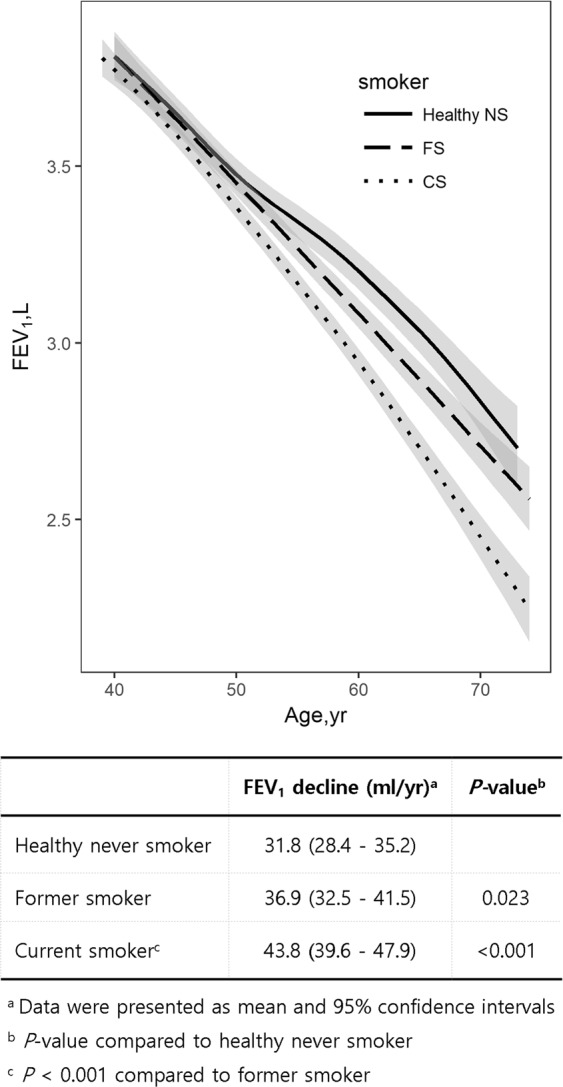
Comparison of the annual mean decline in FEV_1_ among male healthy never smokers (n = 521), former smokers (with and without OLD; n = 681), and current smokers (with and without OLD; n = 1138). Current smokers (43.8 mL) showed the fastest annual rate of decline in FEV_1_, followed by former smokers (36.9 mL) and healthy never smokers (31.8 mL), sequentially. Abbreviations: OLD, obstructive lung disease; CI, confidence interval; CS, current smoker; FEV_1_, forced expiratory volume in 1 second; FS, former smoker; NS, never smoker.

Figure 3 compares the decline in FEV_1_ among male healthy never smokers, former smokers with and without OLD, and current smokers with and without OLD. The baseline characteristics of the subjects are shown in Supplementary Table 1. The annual rate of decline in FEV_1_ was the fastest for current smokers (43.8 mL; 95% CI, 39.6–47.9; *P* < 0.001 vs. healthy never smokers and former smokers), while it was more rapid for former smokers (36.9 mL; 95% CI, 32.5–41.5; *P* = 0.023) than for healthy never smokers (31.8 mL; 95% CI, 28.4–35.2; Fig. 3). It also tended to be more rapid for current smokers (32.0 mL; 95% CI, 25–2–38.8) than for healthy never smokers (27.2 mL; 95% CI, 26.2–28.2; *P* = 0.165).

**Figure 4:**
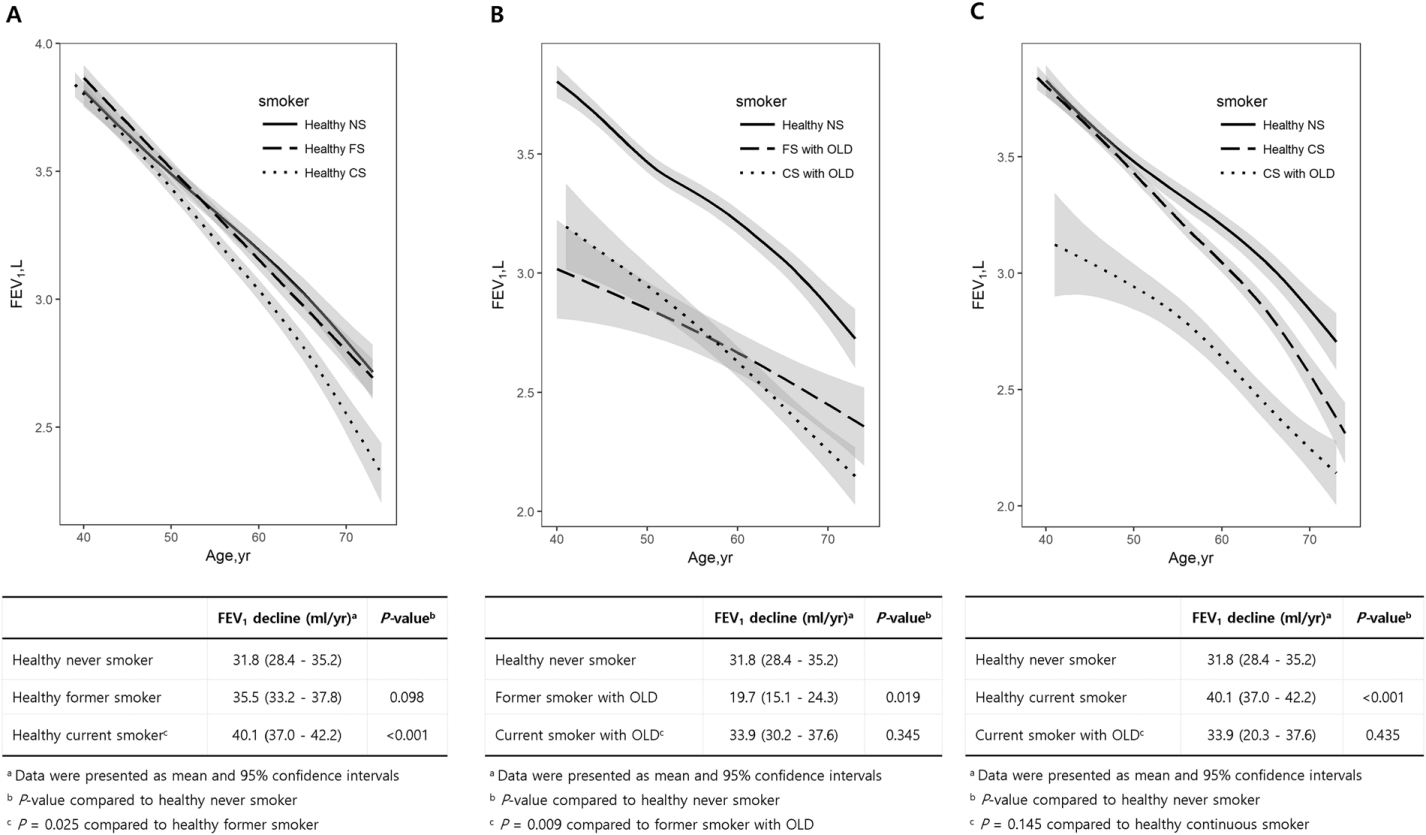
Comparison of the annual mean decline in FEV_1_ according to the smoking status and presence or absence of OLD in men. Notes: (**A**) Comparison of the annual mean decline in FEV_1_ among healthy never smokers (n = 521), healthy former smokers (n = 595), and healthy current smokers (n = 968). The annual rate of decline in FEV_1_ is the fastest for healthy current smokers (40.1 mL). (**B**) Comparison of the annual mean decline in FEV_1_ among healthy never smokers (n = 521), former smokers with OLD (n = 86), and current smokers with OLD (n = 170). The annual rate of decline in FEV_1_ is the slowest for former smokers with OLD (19.7 mL). (**C**) The annual mean decline in FEV_1_ is comparable between healthy current smokers (n = 968) and current smokers with OLD (n = 170). The annual rate of decline in FEV_1_ is faster in healthy current smoker than in healthy never smoker (40.1 mL vs. 31.8 mL; *P* < 0.001). Abbreviations: CI, confidence interval FEV_1_, forced expiratory volume in 1 second; OLD, obstructive lung disease.

Figure 4 shows the comparisons among male healthy never smokers, healthy former smokers, healthy current smokers, former smokers with OLD, and current smokers with OLD. The baseline characteristics of the subjects are shown in Supplementary Table 2. Figure 4A compares the decline in FEV_1_ among male healthy never smokers, healthy former smokers, and healthy current smokers. The annual rate of decline in FEV_1_ was the fastest for healthy current smokers (40.1 mL; 95% CI, 37.0–42.2; *P* < 0.001 vs. healthy never smokers; *P* = 0.025 vs. healthy former smokers), while it was statistically indistinguishable between healthy former smokers (35.5 mL; 95% CI, 33.2–37.8 mL) and healthy never smokers (*P* = 0.098).

Figure 4B compares the decline in FEV_1_ among male healthy never smokers, former smokers with OLD, and current smokers with OLD. The annual rate of decline in FEV_1_ was significantly more rapid for current smokers with OLD (33.9 mL; 95% CI, 30.2–37.6) than for former smokers with OLD (19.7 mL; 95% CI, 15.1–24.3; *P* = 0.009), while there was no significant difference between healthy current smokers and current smokers with OLD (Fig. 4C).

## Discussion

In the present study, the annual decline in lung function was estimated for a population-based cohort in Korea. Healthy men and those with OLD who were current smokers showed a significantly increased rate of decline in FEV_1_ than did their former smoker counterparts. The rate of decline in FEV_1_ was similar between former smokers and never smokers among men without OLD and between male healthy current smokers and male current smokers with OLD.

Studies of the decline in lung function in population-based cohorts have been previously reported^25,26^. The mean decline in FEV_1_ in healthy never smokers in our study was similar to that in the European Community Respiratory Health Survey (ECRHS)^26^. We found annual mean FEV_1_ declines of 31.8 mL for men and 27.2 mL for women, while the ECRHS reported respective decreases of 32 mL and 24 mL^26^. Although the participants in our study were older than those in the ECRHS, they were taller on average. The average annual decline in FEV_1_ in current smokers in our study was consistent with that in the Swiss Study on Air Pollution and Lung Diseases in Adults, where the annual mean decline in FEV_1_ was 43.8 mL for male current smokers and 34.7 mL for their female counterparts (43.7 mL for men and 32.0 mL for women in our study)^25^. Omori *et al*. reported similar declines in FEV_1_ in the Japanese male population stratified by age and the smoking status^24^.

Several previous reports have analysed the relationship between declining lung function and smoking status in healthy subjects^25,27–33^. The natural history of COPD has been determined mainly from the study by Fletcher and Peto, who evaluated the decline in lung function in 1,136 working men aged 35–59 years over an 8-year follow-up period^4^. On the basis of a limited number of observations, these authors proposed that the decline in FEV_1_ was significantly accelerated in a subgroup of smokers^4^. Kohansal *et al*. found that current smoking increased the rate of decline in FEV_1_ in a large cohort of men and women^31^. Our study confirmed that tobacco smoking increased the rate of decline in lung function in men. However, the number of female current smokers in our study was too small for the detection of a statistically significant difference between current smokers and healthy never smokers.

Fletcher and Peto considered that the rate of decline in FEV_1_ would normalize after smoking cessation at any age^4^. Many studies have reported beneficial effects of smoking cessation on lung function decline^27,31,34^. We found that the rate of decline in FEV_1_ in men was not different between healthy never smokers and healthy former smokers. In the study by Kohansal *et al*., smoking cessation had a beneficial effect at any age, although it was more pronounced in earlier quitters^31^. The rate of decline in FEV_1_ in both men and women who smoked but quit before the age of 30 years was similar to that in healthy never smokers^31^.

In the present study, the rate of decline in FEV_1_ in current smokers with OLD was similar to that in healthy never smokers and healthy current smokers. In a population-based cohort from three cities in Latin America, the annual mean post-bronchodilator decline in FEV_1_ was 27 mL for patients with COPD at baseline and 36 mL for those without COPD^32^. A more rapid decline was associated with older age, higher baseline lung function, and smoking at baseline^32^. A greater decline in FEV_1_ in men and patients with better lung function suggests that the decline is proportional to lung size^32,33^. In a study by Ware *et al*., the annual rate of decline was greater for elderly nonsmokers (12.9 mL at the age of 25 years vs. 58.2 mL at the age of 75 years)^35^.

Several studies evaluating the decline in lung function in Korean patients with COPD have been published^36,37^. Kim *et al*. reported that the adjusted annual rate of decline in the postbronchodilator FEV_1_ was 28.3 mL, with no significant difference in the rate of decline in lung function among groups stratified according to the 2014 Global Initiative for Chronic Obstructive Lung Disease (GOLD) guidelines (−34.4 ± 7.9 [group A], −26.2 ± 9.4 [group B], −22.7 ± 16.0 [group C], and −24.0 ± 8.7 mL/year [group D])^36^. The decline that we report in healthy never smokers was more rapid than that in former smokers with OLD, and slower than that in current smokers with OLD. This discrepancy may be attributable to baseline FEV_1_ differences between the two studies; the airflow limitation in smokers with OLD in the present study was milder than that in the GOLD A group in the study by Kim *et al*. Small number of former smokers with OLD might not represent real lung function decline of former smokers with OLD. However, in the group of former smoker with OLD, absolute FEV_1_ at 40 years was lower than in the group of healthy never smoker. Therefore, the amount of decline of FEV_1_ might be less in the group of former smoker with OLD because healthy never smokers have more to lose than former smokers with OLD in early stage. In another study, the annual post-bronchodilator FEV_1_ declined more rapidly in older patients with COPD (25.0 mL for patients older than 67 years vs. 13.0 mL for younger patients)^37^. However, the rate of decline in lung function in the healthy population in Korea has not been reported. We investigated the effect of smoking and smoking cessation on the normal decline in lung function over time in a large cohort of both men and women, including healthy never smokers.

Our study has some strengths and potential limitations. The main strengths include its prospective design and the inclusion of spirometric measurements for a large population-based cohort including both rural and urban communities. However, the study duration was relatively short. Given that half the subjects were aged 40–49 years, they would have still been younger than 55 years after a mean follow-up period of 3.8 years. However, the Ansung-Ansan cohort study is ongoing, and we hope to analyse the data further with a longer follow-up period. Moreover, we used pre-bronchodilator spirometric values rather than post-bronchodilator values; accordingly, we used OLD for patient stratification instead of COPD. In addition, data concerning smoking cessation were purely based on a questionnaire, with no objective confirmation using a biological method. Self-reporting of the smoking status may be biased by societal factors, because women are less likely to report their smoking status because of the fear of stigma. Moreover, the duration of smoking in pack-years, and the mean number of years since smoking cessation could not be analysed because of the lack of data. Furthermore, because of the lack of information such as birth weight, we could not evaluate the impact of lung underdevelopment on lung function. In addition, the number of women in all groups except for the healthy never smoker group was very small. Because the analysis of these groups might result in very unreliable estimates, only healthy never smokers were analysed. Finally, we could not exclude the participants with a restrictive pattern on spirometry.

In conclusion, the present study demonstrated the rate of decline in lung function over time in a large community-based Korean cohort. The findings suggest that continued smoking accelerates the decline in lung function, regardless of the presence of OLD. This underscores the importance of smoking cessation in the Korean population.

## Methods

### Study populations

The ongoing Ansung-Ansan cohort study was initiated in 2001 and supported from the National Genome Research Institute (Korea Centers for Disease Control and Prevention). It is a part of the Korean Genome and Epidemiology Study, which is based on a series of large community-based epidemiological surveys investigating chronic disease in Koreans. Further information on the study design and protocols have been published previously^38,39^.

From Ansung-Ansan cohort I (2001–2002), 8,842 subjects aged 40–69 years with valid lung function measurements and no previous history of asthma, bronchiectasis, or lung destruction, were included. Follow-up examinations and surveys are performed biennially (Fig. 1). We analysed data from a baseline survey and two subsequent biennial surveys (I–III: 2001–2006). Of the 8,842 subjects in the Ansung-Ansan cohort I, 7,111 who underwent pulmonary function testing ≥2 times during surveys I–III were used as the study population in the present research. Valid data concerning the smoking history were available for 5,865 of the 7,111 subjects.

The Korea Centers for Disease Control and Prevention obtained written informed consent from all participants on collection of the data, and the Institutional Review Board of Severance Hospital approved the present study protocol (4-2016-0458). All methods were performed in accordance with the approved protocol and with the relevant guidelines and regulations.

### Spirometry

Lung function was assessed using spirometry (VMAX2130, SensorMedics Corporation, Yorba, CA, USA) at baseline and the first and second follow-up visits. Spirometry was performed according to American Thoracic Society/European Respiratory Society guidelines^40^.

### Definition

Individuals who declared that they had never smoked before the study were considered never smokers, those who declared that they had smoked but stopped before the study were considered former smokers, and those who reported active smoking during the study period were considered current smokers. OLD was defined by a pre-bronchodilator FEV_1_/forced vital capacity ratio of <0.7. Individuals without OLD were considered healthy subjects.

### Statistical analysis

We stratified participants into groups according to the smoking status (never smoker, former smoker, and current smoker) at cohort inception and the presence or absence of OLD. Subjects who did not respond to the questionnaires for the smoking history were excluded, while those with consistent reporting of the smoking history were included and categorized into never, former, and current smokers. Subjects whose smoking status changed from current smoker to former smoker during the study period were categorized as former smokers. We then determined the rate of decline in FEV_1_ over time according to the smoking status, sex, and OLD status in subjects for whom two or more spirometry measurements were available.

The FEV_1_ values measured at each assessment were analysed using linear mixed models. We included age, sex, the body mass index, height, the smoking status, and the interaction of age and smoking as covariates. The model for all subjects *i* at time point *j* was as follows:

$$\,\begin{array}{rcl}FE{V}_{{1}_{ij}} & = & {{\rm{\beta }}}_{0}\,+\,{\beta }_{1}ag{e}_{ij}\,+\,{\beta }_{2}se{x}_{i}+{\beta }_{3}BM{I}_{ij}\,+\,{\beta }_{4}smoking\,status+{\beta }_{5}age\cdot smoking\,status\,+\,{b}_{i}\,+\,{{\epsilon }}_{ij},\\  &  & where\,{({\epsilon }_{{{\rm{ij}}}_{1}},\mathrm{..},{{\epsilon }}_{i{j}_{n}})}^{t} \sim MVN(0,\Sigma ),\,{b}_{i} \sim iid\,MVN(0,{\sigma }^{2}),\end{array}$$

while the model for female or male sex was as follows:

$$\begin{array}{rcl}FE{V}_{{1}_{ij}} & = & {{\rm{\beta }}}_{0}\,+\,{\beta }_{1}ag{e}_{ij}+{\beta }_{2}BM{I}_{ij}\,+\,{\beta }_{3}smoking\,status\,+\,{\beta }_{4}age\cdot smoking\,status\,+\,{b}_{i}\,+\,{{\epsilon }}_{ij},\\  &  & where\,{({\epsilon }_{{{\rm{ij}}}_{1}},\mathrm{..},{{\epsilon }}_{i{j}_{n}})}^{t} \sim MVN(0,\Sigma ),\,{b}_{i} \sim iid\,MVN(0,{\sigma }^{2}).\end{array}$$

Generalized additive mixed models^41^ were used to determine the best fit for the decline in FEV_1_ over time, and all figures were generated from these models. All statistical analyses were performed using R version 3.4.3 (R Foundation for Statistical Computing, Vienna, Austria). For the linear mixed models and the generalized linear mixed models, the “nlme” and “mgcv” packages, respectively, were used.

## Supplementary information

Supplementary data
