# Supplementary material for: Longitudinal decline in lung function: a community-based cohort study in Korea
Source: Sci Rep. 2019 Sep 20;9:13614. doi: 10.1038/s41598-019-49598-9 (PMC6754397; doi:10.1038/s41598-019-49598-9)
Supplement: Supplementary file 1 — Supplementary data [file 41598_2019_49598_MOESM1_ESM.pdf]

Longitudinal decline in lung function: a community-based cohort study in Korea

Ah Young Leem[1¶], Boram Park[2¶], Young Sam Kim[1], Joon Chang[1], Sungho Won[2†], Ji Ye Jung[1†]*

[1]Division of Pulmonology, Department of Internal Medicine, Institute of Chest Disease, Severance Hospital, Yonsei University College of Medicine, Seoul, Republic of Korea

[2]Department of Epidemiology and Biostatistics, School of Public Health, Seoul National University, Seoul, Korea

[¶]These authors contributed equally to this work as first authors.

†These authors contributed equally to this work as corresponding authors.

***Corresponding Authors**: Ji Ye Jung, M.D., Ph.D.

Assistant Professor, Division of Pulmonology, Department of Internal Medicine, Institute of Chest Disease, Severance Hospital, Yonsei University College of Medicine

50-1 Yonsei-ro, Seodaemun-gu, Seoul 120-752, Republic of Korea

Tel.: 82-10-3141-2576

Fax: 82-2-393-6884

E-mail: STOPYES@yuhs.ac

Supplementary data

Supplementary Table 1. Baseline characteristics of population categorized by sex and smoking status

Characteristics	Men			Women	
	**Healthy never smoker (n=521)**	**Former smoker (n=681)**	**Current smoker (n=1,138)**	**Healthy never smoker (n=3,297)**	**Current smoker (n=62)**
Age, yr	51.2 ± 8.3	51.5 ± 8.3	50.5 ± 8.3	52.1 ± 8.8	55.2 ± 9.7
Height, cm	166.6 ± 5.7	167.4 ± 5.9	167.2 ± 5.6	154.0 ± 5.5	153.9 ± 4.9
Weight, kg	68.5 ± 8.9	69.9 ± 9.1	67.2 ± 9.6	59.2 ± 8.3	57.6 ± 8.3
BMI, kg/m^2	24.7 ± 2.8	24.9 ± 2.6	24.0 ± 2.9	25.0 ± 3.2	24.3 ± 3.1
Lung function					
FEV_1, liters	3.5 ± 0.5	3.4 ± 0.6	3.3 ± 0.6	2.5 ± 0.5	2.4 ± 0.5
FEV_1, % of predicted value	110.8 ± 14.2	107.5 ± 14.9	105.3 ± 15.1	117.9 ± 16.6	115.5 ± 19.7
Follow-up duration					
2 years	158 (30.3)	0 (0.0)	508 (44.6)	1,268 (38.5)	32 (47.8)
4 years	363 (69.7)	681 (100.0)	630 (55.4)	2,029 (61.5)	35 (52.2)

Notes: Data are presented as number (%) or the mean ± standard deviation**.**

Abbreviations: BMI, body mass index; FEV_1, forced expiratory volume in 1 second.

Supplementary Table 2. Baseline characteristics of men categorized by lung function and smoking status

Characteristics	Healthy			Obstructive Lung Disease	
	Never smoker (n=521)	Former smoker (n=595)	Current smoker (n=968)	Former smoker (n=86)	Current smoker (n=170)
Age, yr	51.2 ± 8.3	50.6 ± 7.9	49.0 ± 7.7	57.7 ± 7.8	58.6 ± 7.1
Height, cm	166.6 ± 5.7	167.6 ± 5.9	167.5 ± 5.6	165.9 ± 5.8	165.7 ± 5.1
Weight, kg	68.5 ± 8.9	70.2 ± 9.1	68.0 ± 9.6	67.6 ± 8.9	62.5 ± 8.3
BMI, kg/m^2	24.7 ± 2.8	24.9 ± 2.6	24.2 ± 2.9	24.5 ± 2.6	22.8 ± 2.6
Lung function					
FEV_1, liters	3.5 ± 0.5	3.5 ± 0.5	3.5 ± 0.6	2.7 ± 0.5	2.7 ± 0.5
FEV_1, % of predicted value	110.8 ± 14.2	109.6 ± 13.8	107.6 ± 14.1	93.1 ± 14.7	92.7 ± 14.1
Follow-up duration					
2 years	158 (30.3)	0 (0.0)	402 (41.5)	0 (0.0)	106 (62.4)
4 years	363 (69.7)	595 (100.0)	566 (58.5)	86 (100.0)	64 (37.6)

Notes: Data are presented as number (%) or the mean ± standard deviation**.**

Abbreviations: BMI, body mass index; FEV_1, forced expiratory volume in 1 second.
